# Estimation of synthetic accessibility score of drug-like molecules based on molecular complexity and fragment contributions

**DOI:** 10.1186/1758-2946-1-8

**Published:** 2009-06-10

**Authors:** Peter Ertl, Ansgar Schuffenhauer

**Affiliations:** 1 Novartis Institutes for BioMedical Research, Novartis Campus, CH-4002 Basel, Switzerland

## Abstract

**Background:**

A method to estimate ease of synthesis (synthetic accessibility) of drug-like molecules is needed in many areas of the drug discovery process. The development and validation of such a method that is able to characterize molecule synthetic accessibility as a score between 1 (easy to make) and 10 (very difficult to make) is described in this article.

**Results:**

The method for estimation of the synthetic accessibility score (SAscore) described here is based on a combination of fragment contributions and a complexity penalty. Fragment contributions have been calculated based on the analysis of one million representative molecules from PubChem and therefore one can say that they capture historical synthetic knowledge stored in this database. The molecular complexity score takes into account the presence of non-standard structural features, such as large rings, non-standard ring fusions, stereocomplexity and molecule size. The method has been validated by comparing calculated SAscores with ease of synthesis as estimated by experienced medicinal chemists for a set of 40 molecules. The agreement between calculated and manually estimated synthetic accessibility is very good with *r*^2 ^= 0.89.

**Conclusion:**

A novel method to estimate synthetic accessibility of molecules has been developed. This method uses historical synthetic knowledge obtained by analyzing information from millions of already synthesized chemicals and considers also molecule complexity. The method is sufficiently fast and provides results consistent with estimation of ease of synthesis by experienced medicinal chemists. The calculated SAscore may be used to support various drug discovery processes where a large number of molecules needs to be ranked based on their synthetic accessibility, for example when purchasing samples for screening, selecting hits from high-throughput screening for follow-up, or ranking molecules generated by various *de novo *design approaches.

## Background

The assessment of synthetic accessibility (SA) of a lead candidate is a task which plays a role in lead discovery regardless of the method the lead candidate has been identified with. In the case of a *de novo *designed molecule the experimental validation of its activity requires synthesis of the compound. In the case of experimental or virtual screening exploration of the SAR around the hit, synthetic access to the chemotype is required as well. The more difficult the synthesis of the lead candidate is, the more time and resources are needed for the exploration of this particular area of chemical space. Lead candidates are normally prioritized according to criteria such as drug-likeness [1,2], natural-product likeness [3], predicted activity or freedom to operate with respect to intellectual property. Since sooner or later in the drug discovery process the candidates will be ranked, or even eliminated by their synthetic accessibility, it is desirable to include this aspect into the prioritization of compounds early on. When compounds are purchased from off-the-shelf catalogues in order to augment the screening library, compounds likely to fail later on because of problems with their synthetic tractability may be removed already at this stage. Also in the selection of follow-up candidates from large primary screening results, prioritization by synthetic accessibility can ensure that compounds chosen for validation in dose-response experiments are less likely to be later rejected based on problems with their synthesis. In these two cases, the compounds that are to be validated exist, which means that chemical synthesis must in principle be feasible despite possible complications. When chemical structures are constructed during the *de novo *design process, one cannot take for granted that the chemical synthesis of such compounds is feasible at all. Therefore it is even more important to estimate whether these compounds can be synthesized with reasonable effort. While experienced chemists are able to estimate synthetic accessibility of individual compounds, performing this estimation for large numbers of compounds requires computational methods.

Several computational approaches to assess synthetic accessibility of molecules exist [4]. They may be roughly divided into two groups: complexity-based and retrosynthetic-based. Complexity-based methods use sets of rules to estimate complexity of target structures (features like presence of spiro-rings, non-standard ring fusions, or large number of stereocenters) which is then directly related to SA. The second group of methods is based on the full retrosynthetic approach when the complete synthetic tree leading to the molecules needs to be processed. Such a procedure is quite time consuming, because the size of the synthetic tree grows exponentially with the number of required steps. Additionally, retrosynthetic methods rely on reaction databases as well as lists of available reagents, which both need to be kept up-to-date. This high requirement on maintenance is probably one of the reasons why methods for estimation of SA based on the retrosynthetic approach have been developed mainly by large academic teams (for example group of Prof. Gasteiger at Erlangen University with the WODCA system [5] or group of Prof. Johnson at Leeds University with the SPROUT/CAESA program [6]).

The major problem when developing methods for estimation of SA is the validation of results. It is not straightforward to extract synthetic complexity out of the protocol describing molecule synthesis. While the overall yield over the sequence of synthetic steps gives some information, this depends also on the effort which has been undertaken to optimize the process; and if only low amounts are needed for initial experiments, then a non-optimal synthesis is tolerable. Another possible measure of synthetic accessibility of a molecule could be its price in catalogues of chemical providers. The price, however, depends on too many factors not related to SA (for example novelty of the reagent, demand, packaging, marketing issues) to be relied on as an objective measure of SA. We were not able to get any reasonable correlation between normalised catalogue price and various structural descriptors for a large set of reagents. The total cost of production of pharmaceutical substances, where the whole process is highly optimized concerning the cost of goods and manufacturing expenses, would be probably the most useful parameter in this respect, but unfortunately this type of data is one of the most guarded secrets in the pharmaceutical industry.

Therefore currently the only way to assess the performance of the calculated synthetic accessibility score is to rely on a ranking done by experienced medicinal chemists.

Several studies focused on performance of chemists in ranking molecules or estimating their synthetic accessibility. In the work of Takaoka *et al*. [7] 5 chemists ranked 3980 molecules according to their ease of synthesis into three categories: easy, possible and hard. Correlation coefficients between scores assigned by various chemists were in the range 0.40 to 0.56 with an average 0.46. The authors concluded, however, that the models based on the average of chemist estimations may be useful for classification of molecules. Baber and Feher [4] described an experiment where 8 medicinal chemists scored 100 drug-like compounds according to their ease of synthesis. The mean absolute error in chemists' estimations was around 10%, for some compounds, however, there was a variation of up to 70%. In the study of Lajiness *at al*. [8], 13 chemists reviewed sets of 2000 diverse compounds containing also a common set of 250 compounds, with the goal of removing those that are unacceptable for any reason (too complex, having too complicated synthesis, unsuitable for launching a drug discovery campaign etc): the objective was to see the consistency of chemists in picking "bad" molecules. The study has shown that chemists are not very consistent in their rejection of compounds: only 24% of the compounds rejected by one chemist were also rejected by another. Boda *et al*. [9] asked 5 chemists to rank 100 molecules selected randomly from the Journal of Medicinal Chemistry according to their ease of synthesis. The chemists seemed to agree on synthetic accessibility for very simple and quite complex molecules; in the middle range, however, larger divergence was observed. The agreement among chemists was acceptable with correlation coefficients in the range 0.73 – 0.84. The ranks entered by chemists have been then used to train the synthetic accessibility score function described in the publication.

All these studies indicate that even experienced chemists differ in their estimations of ease of synthesis. This, of course, is nothing surprising. Chemists have different backgrounds, different areas of research (medicinal chemists, natural product chemists, chemists working in combinatorial synthesis, etc.) or experience based on projects they have been working on. Therefore, to use ranks assigned by chemists as a measure of SA, a consensus score based on several estimations is required. The situation is additionally complicated by the fact that the ease of synthesis for a particular molecule is not a constant. It evolves within time as a consequence of introduction of new synthetic methods and availability of new reagents and building blocks. For example, an introduction of methods like carbon-carbon coupling reactions, sophisticated organometallic catalysts or use of enzymes in organic synthesis allows currently relatively easy synthesis of molecules, which would be very difficult to make just a decade ago [10].

### Calculation of Synthetic Accessibility Score

The goal of the present study was to develop a method for estimation of SA which could be used in various drug discovery activities. The fact that the method should be able to process very large numbers of molecules (several millions when making a selection from large commercial catalogues or processing virtual libraries), as well as a decision not to rely on comprehensive databases of reactions and reagents (with the related maintenance hurdle) clearly favored implementation of a method based on molecular complexity. Pure complexity-based approaches, however, have known deficiencies: they do not take into account easy availability of complex reagents, which allows us to introduce some complex features to molecules relatively easily [6], neither the fact that some simple reactions can produce quite complex structures (condensation reactions, cycloadditions, various cyclizations). To account for this deficit of a pure complexity-based approach we have decided to implement a method which would be a compromise between fast complexity-based, and resource-intensive full retrosynthetic approaches. In addition to several standard rules identifying known synthetically problematic molecular features, we wanted to capture also the "synthetic chemistry knowledgebase" by analyzing common substructures in a very large number of already synthesized molecules. For this purpose, a representative subset of molecules from the PubChem database [11] was used. PubChem contains currently 37 million unique molecules including common drugs and agrochemicals, structures extracted from patents, and large numbers of samples from numerous compound providers. One million molecules representatively selected from PubChem served as a training set to identify common (and therefore one can assume also easy to make) structural features. Our approach is similar to those presented by Boda and Johnson [12] who based their estimation of molecular complexity on a set of simple fragments collected from a database of drug-like molecules. Our fragment approach differs, however, in using different types of fragments, as well as by a different method to calculate fragment contributions.

The synthetic accessibility score – SAscore in our approximation is calculated as a combination of two components:

The fragmentScore, as already mentioned, was introduced to capture the "historical synthetic knowledge" by analyzing common structural features in a large number of already synthesized molecules. The score is calculated as a sum of contributions of all fragments in the molecule divided by the number of fragments in this molecule. The database of fragment contributions has been generated by statistical analysis of substructures in the PubChem collection as described in the following section.

**Figure 1:**
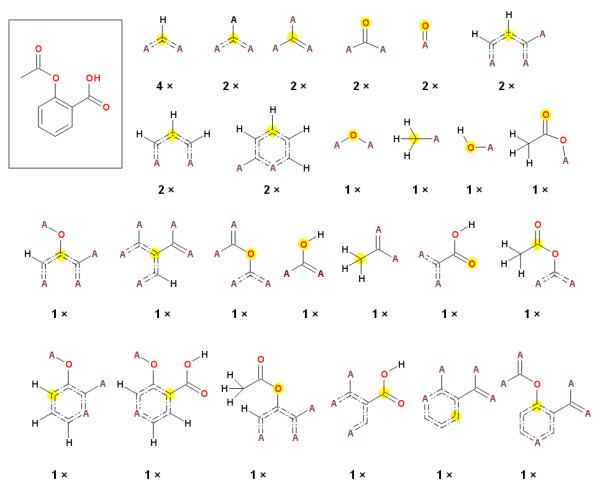
**Substructures obtained by fragmentation of Aspirin, the "A" represents any non-hydrogen atom, "dashed" double bond indicates an aromatic bond, number below the fragment indicates the count of this substructure in the molecule and the yellow circle marks the central atom of the fragment**.

934,046 representative molecules from the PubChem database were fragmented. Extended connectivity fragments (ECFC_4# fragments) as implemented in Pipeline Pilot [13] were used. This type of fragment includes a central atom, as well as several levels of neighbors connected to the central atom by one to three bonds. The type of atoms in the last level is not specified and they all are marked as "star" atoms only. To illustrate this procedure the fragments generated for Aspirin are shown in Figure 1 together with their frequency of occurrence. The size of fragments we used has been chosen intentionally to be quite large to capture also information about rings, and relative positions of multiple substitution points on the rings (information which is very important for estimation of SA [12]).

**Figure 2:**
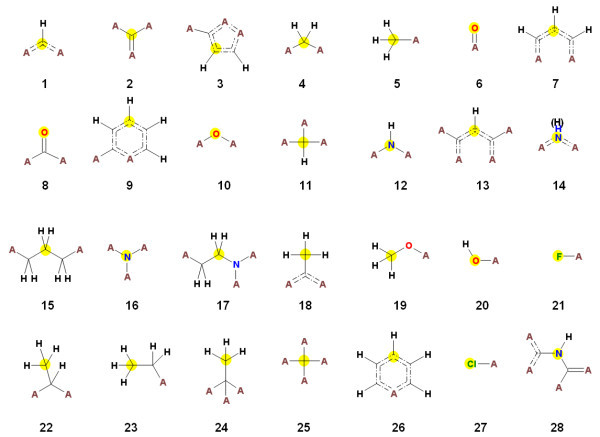
**The most common fragments present in the million PubChem molecules**. The "A" represents any non-hydrogen atom, "dashed" double bond indicates an aromatic bond and the yellow circle marks the central atom of the fragment.

Altogether 605,864 different fragment types have been obtained by fragmenting the PubChem structures. Most of them (51%), however are singletons (present only once in the whole set). Only a relatively small number of fragments, namely 3759 (0.62%), are frequent (i.e. present more than 1000-times in the database). The most common fragments are shown in Figure 2.

**Figure 3:**
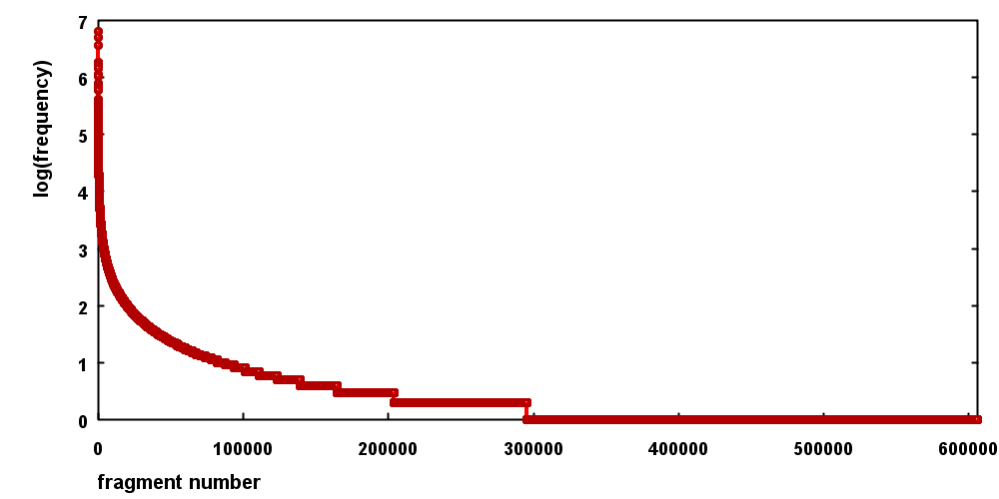
**Frequency distribution of fragments**.

The frequency distribution for the whole fragment set is shown in Figure 3. Based on this distribution the contribution for each fragment has been calculated as a logarithm of the ratio between the actual fragment count and the number of fragments forming 80% of all fragments in the database. As a result the frequent fragments have positive scores and less frequent fragments have negative scores. The whole approach is based on a simple assumption that the fragment frequency is related to their synthetic accessibility – substructures which are easy to prepare are present in molecules quite often, those which are difficult to synthesize or are unstable are rare.

**Figure 4:**
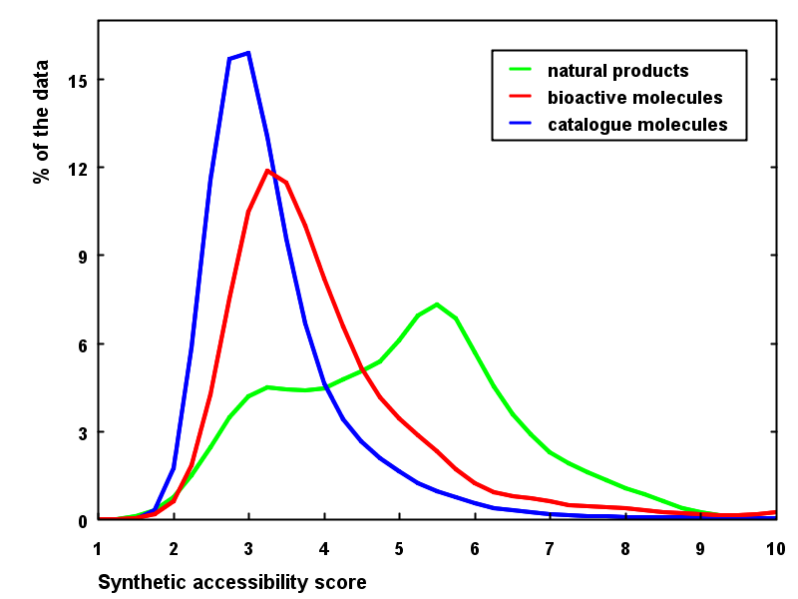
**Distribution of SAscore for natural products, bioactive molecules and molecules from catalogues**.

The complexityScore is simply a number that characterizes the presence of complex structural features in the molecules. It is calculated as a combination of ringComplexityScore, stereoComplexityScore, macrocyclePenalty and the sizePenalty. This is close to the way in which chemists assess molecular complexity themselves. The ringComplexityScore characterizes complexity of ring systems in molecules (which is probably the most important factor influencing molecular complexity) based on detection of spiro rings and ring fusions. The stereoComplexity penalizes molecules with many potential stereo centers. The penalty for presence of macrocycles increases molecular complexity when rings of size > 8 are present in the molecule. These factors are calculated as:

After subtracting the complexity penalty from the fragment score, the result (normally in the range -4 (worst) to 2.5 (best)) is multiplied by -1 and scaled to be between 1 and 10 to provide simply a value which is easier to interpret. In the rest of this publication we will use the term SAscore for this value. Molecules with the high SAscore (say, above 6, based on the distribution of SAscore shown in the Fig. 4) are difficult to synthesize, whereas, molecules with the low SAscore values are easily synthetically accessible. We did not make any attempts to find optimal weights of complexity and fragment contributions (as was done for example in the study [9] where these weights have been optimized to fit the ranks assigned by chemists). Our dataset (40 molecules) was relatively small and any optimization of parameters would probably lead to overfitting. The complexity parameters act in this sense rather as "indicator variables" increasing the SAscore for molecules containing synthetically problematic features.

To illustrate the performance of the new SAscore, its distribution for 100,000 synthetic molecules from catalogues of commercial compound providers (not used in the training process), 100,000 bioactive molecules randomly selected from the WDI [14] and MDDR [15] databases and 100,000 natural products from the Dictionary of Natural Products [16] is shown in Figure 4. The graph is consistent with the common presumption that natural products are much more difficult to synthesize than "standard" organic molecules. Bioactive molecules have their SAscore somewhere in the middle between these two sets. This graph should provide some feeling about the meaning of the score and how it is distributed in different molecular data sets.

### SAscore Implementation

To make the SAscore as broadly available as possible at Novartis, we implemented the algorithm in the Pipeline Pilot environment [13]. PipelinePilot protocols are used routinely at Novartis to support various drug discovery activities. The heart of the calculation protocol is the "SAscore Calculator" component, which is a custom component written in PERL, where the actual calculation of the score described in the previous section is implemented. The speed of the protocol is sufficient to process large datasets; SAscore for 100,000 molecules may be calculated in about 3 minutes.

The implementation using other cheminformatics toolkits, however, should be straightforward. Access to simple molecular characteristics such as molecule size, number of stereocenters, presence of macrocycles etc. is provided easily by several free cheminformatics toolkits [17]. Also the generation of atom-centered fragments should not be complicated. Actually the initial prototype implementation of this algorithm has been done by using the Molinspiration molecular processing engine [18] using the HOSE type fragments [19] implemented there, and the results were practically identical to those of PipelinePilot implementation.

### Validation of Synthetic Accessibility Score

**Figure 5:**
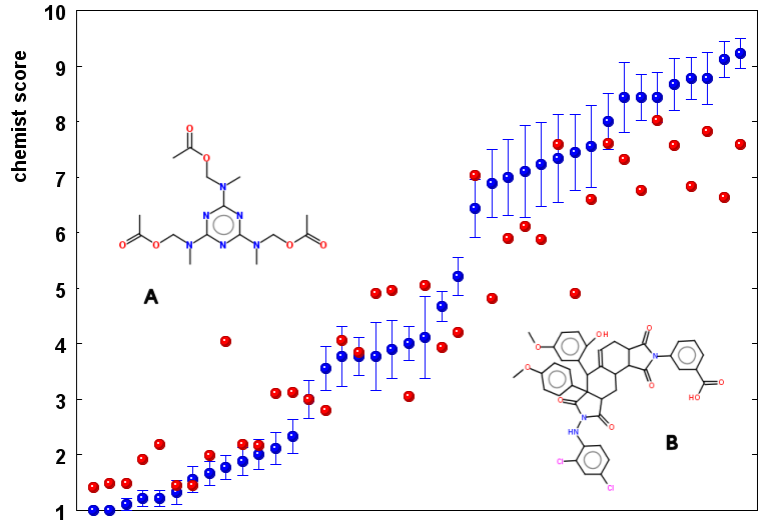
**Average of chemist ranks for 40 test molecules (blue) compared with the computed SAscore (red)**. Error bars on blue points indicate standard error of mean of estimations by 9 chemists.

As mentioned in the introduction, it is not easy to validate the performance of the synthetic accessibility score, because there are no experimental measures or objective molecular characteristics we can compare it to. In order to validate our algorithm we decided therefore to compare the SAscore with an "ease of synthesis" ranking assigned by synthetic chemists. For this purpose 40 molecules were selected randomly from the PubChem database in such a way that the whole range from small to large molecules was covered. Stereochemistry information was discarded, because it is not used directly in the calculation protocol. The number of possible stereoisomers, however, is used in the generation of the complexity part of the score, so the stereocomplexity is captured in this way. Nine Novartis chemists, with long experience in various medicinal chemistry projects, were asked to rank these molecules. To make the ranking process easy, a simple web interface was prepared where all 40 molecules were displayed along with a menu which allowed selection of a score between 1 and 10. The "chemist score" we use in the rest of this article is simply an average of 9 scores entered by chemists (Figure 5). The SMILES codes, chemist scores and the calculated SAscores for all 40 molecules are available as Additional file 1. We want to stress here that this validation experiment was performed only after the development and implementation of the SAscore protocol had been completely finished, and therefore the results could not be used in any way to "tune" the calculation algorithm.

The agreement among chemists in their rankings is quite good, the *r*^2 ^ranges between 0.450 and 0.892 with the average *r*^2 ^for all "chemist pairs" being 0.718. For a few molecules, however, scores by some chemists differ by 6 or more ranks, and for 7 molecules out of 40 the standard deviation is above 2. Average standard deviation for all 40 molecules is 1.23 and average standard error of mean (shown for all molecules in Figure 5 as error bars) is 0.41. The chemists seem to agree on scores for very simple and very complex molecules better than for structures in the middle region (as mentioned already in [9]). Our results are consistent with the outcome of previous studies, indicating that in order to use ranking by chemists as a reference, one has to use the average of several estimations that smoothes somehow the high individual variation.

**Figure 6:**
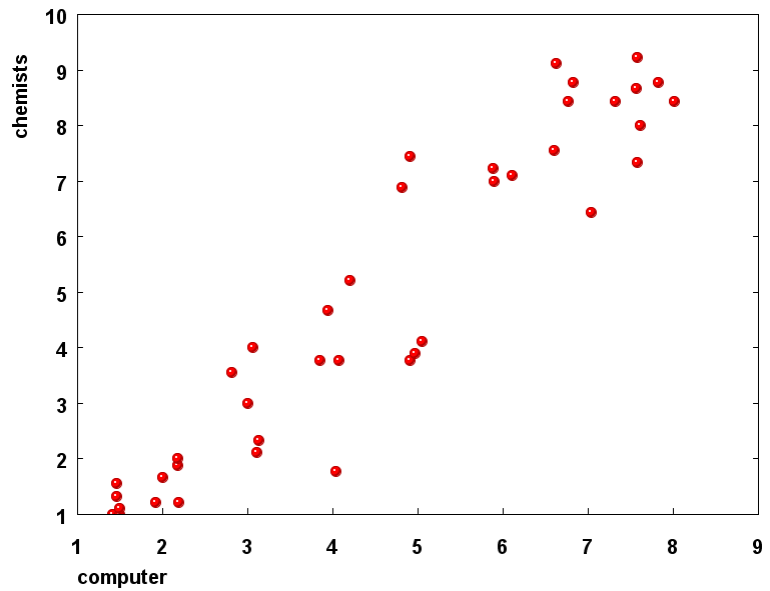
**Correlation of calculated SAscore and average chemist estimation for 40 molecules (*r*^2 ^= 0.890)**.

The correlation between calculated SAscore and the average of chemist ranks is shown in Figure 6. The agreement between these two values is very good, with *r*^2 ^= 0.890 (which means that 89% of variation in the synthetic accessibility as seen by chemists is explained by the SAscore), standard deviation is 0.742. When the SAscore is separated into its two components, the complexityScore provides also very good correlation with the chemist rank (*r*^2 ^= 0.872), while the fragmentScore correlates with the chemist score with *r*^2 ^= 0.628. The chemist score correlates also highly with molecule size (*r*^2 ^for correlation with the number of atoms is 0.688). This correlation, however, is somehow artificial, caused mostly by the fact that our set contains several relatively small molecules, and on the other side also rather large molecules, about which, as already discussed, chemists agree very well on. When only molecules in the middle range (molecular weight between 250 and 550) are considered, the correlation with the molecule size is much lower, (*r*^2 ^= 0.459), while correlation between chemist score and SAscore is still good (*r*^2 ^= 0.803).

We are, of course, aware of the fact that our set containing only 40 molecules is not large enough to draw too general conclusions from the results. The sole purpose of this exercise was to check whether the calculated SAscore correlates with assessment of ease of synthesis by chemists. The data presented here clearly indicates good support for validity of SAscore with both its components (molecular complexity and fragment score) being important for its good performance.

## Discussion

Particularly large differences between chemists and computers could be seen for molecules A and B, both shown in Figure 5. A is a highly symmetrical molecule, which makes synthesis easier, but this factor is not considered when computing the SAscore. We plan therefore to introduce recognition of molecule symmetry in the next version of our SAscore.

Another example where the chemist score and SAscore differ significantly is structure B. In this case chemists overrate the complexity of synthesis. On a first look, the molecule with a central scaffold consisting of 4 fused aliphatic rings indeed looks large and complex. When checking PubChem, however, more than 39,000 molecules with this particular central scaffold can be found. This system may be actually easily synthesized by a sequence of Diels-Alder reactions from simple starting materials [20]. This example nicely illustrates how the fragment score can recognize easy to make substructures even without the necessity to rely on the reaction databases.

In order to get a better understanding of the fragment contributions in the SAscore method, it is helpful to study the most common fragments depicted in Figure 2. They can be grouped into three general groups. The first group consists of frequent side chains. Fragments 5 (methyl), 19 (methoxy), 20 (hydroxy), 21 (fluoro), 23 (ethyl) and 27 (chloro) belong into this category. Fragments 18 and 22 encode a methyl group in a specific environment: attached to an aromatic ring and to an aliphatic carbon. Fragment 26 describes a 6-membered aromatic ring with maximally one substituent and maximally one heteroatom, which must be identical with the substitution site. With the exception of the relatively rare pyridinium group, the simple phenyl group shows this pattern, and therefore 26 can be also counted as a typical side chain fragment. These side chain fragments are also among the most frequent substituents identified in [21]. It is worth noting that many simple, mono-substituted 5-ring hetero-aromatics often used as side chains, such as thiophene, furane, or pyrrole, share fragment 3 regardless of whether substitution is in position 2 or 3. These side-chains are typically available for all types of building blocks and, with the exception of the hydroxyl group, do not generally interfere with most chemical linkage reactions used in parallel synthesis.

Another group of fragments is directly related to these typical linkage reactions. Each molecule synthesized with one of the linkage reactions listed in Table 1 contains at least two of the most frequent fragments as shown in Figure 2. The comparison of the most common fragments with the RECAP bond cleavage types (Figure 2 in [22]) shows that of the 11 RECAP cleavage types only "olefin", "quarternary nitrogen" and "aromatic nitrogen – aliphatic carbon" are not represented by one of the fragments shown in Figure 2. When comparing the cleavage types with the most common fragments, it is noteworthy that the cleaved bond is not always included in the fragments listed in Figure 2. Fragments containing these bonds are often characteristic for one linkage reaction related to one cleavage type, whereas the most common fragments are those which cover more than a single linkage reaction; for example fragment 8 representing carbonyl groups in general or the even more generic fragment 2 describing a carbon atom with one double and two single bonds to non-H atoms.

**Table 1:** Relation between common linkage reactions and most common fragments shown in Figure 2.

Linkage Reaction	Fragment(s)
**Amide bond formation or Urea formation**	**2, 6, 8, 12 **(from primary amine) or **16 **(from secondary amine), **28 **(only if aniline)
**Sulfonamide formation**	**6, 12 **(from primary amine) or **16 **(from secondary amine)
**Ester formation**	**2, 6, 8, 10**
**Reductive amination**	**4 **(the CH_2 _group from the aldehyde carbon), **12 **(from primary amine) or **16 **(from secondary amine)

A third group of the most common fragments generally represent frequent structural features. Fragments 1, 3, 7, 9, 13 highlight the prevalence of aromatic rings in the space of easily accessible chemistry. Fragment 14 represents any aromatic nitrogen. Usage of piperazine as a linker is represented beside the fragments listed in Table 1 and also by presence of fragment 17.

## Conclusion

A novel methodology to calculate synthetic accessibility score of drug-like molecules has been developed. The method is based on the combination of molecule complexity and fragment contributions obtained by analyzing structures of a million already synthesized chemicals, and in this way captures also historical synthetic knowledge. The method provides good reliability and is sufficiently fast to process very large molecular collections. The performance of the SAscore has been validated by comparing it with the "ease of synthesis" ranks estimated by experienced medicinal chemists, with very good agreement between these two values (*r*^2 ^= 0.890). The application area of the SAscore is to rank large collections of molecules, for example to prioritize molecules when purchasing samples for screening, support decisions in hitlist triaging or rank *de novo *generated structures.

Despite the good performance of the SAscore documented above, we are well aware also of limitations of this method. The SAscore cannot compete with more sophisticated approaches for estimation of synthetic accessibility which reconstruct the full synthetic path, in cases when the results are critical, for example when making decision about selection of a development compound from several candidates. And the ultimate measure for assessing synthetic accessibility of complex organic molecules still remains to be a cumulative experience of skilled medicinal chemists.

## Competing interests

The authors declare that they have no competing interests.

## Authors' contributions

PE (http://peter-ertl.com) developed the SAscore method. AS contributed to the development and discussion and provided indispensable contribution to the development of PipelinePilot SAscore protocol.

## Supplementary Material

Additional file 1 File containing structures of molecules used in the validation process encoded as SMILES, their synthetic feasibility estimated by chemists and calculated SAscores. Click here for file
